# Association of Respiratory Syncytial Virus Infection and Underlying Risk Factors for Death Among Young Infants Who Died at University Teaching Hospital, Lusaka Zambia

**DOI:** 10.1093/cid/ciab466

**Published:** 2021-09-02

**Authors:** Leah S Forman, William Macleod, Lawrence Mwananyanda, Geoffrey Kwenda, Rachel Pieciak, Zachariah Mupila, Caitriona Murphy, Donald Thea, Chilufya Chikoti, Baron Yankonde, Benard Ngoma, Charles Chimoga, Christopher J Gill

**Affiliations:** 1 Boston University School of Public Health, Biostatistics and Epidemiology Data Analytics Center (BEDAC), Boston, Massachusetts, USA; 2 Boston University School of Public Health, Department of Global Health, Boston, Massachusetts, USA; 3 Right to Care—Zambia, Lusaka, Zambia; 4 School of Health Sciences, Department of Biomedical Sciences, University of Zambia, Lusaka, Zambia

**Keywords:** RSV, infant mortality, Zambia, risk factors

## Abstract

**Background:**

Respiratory syncytial virus (RSV) is a leading cause of acute lower respiratory tract infections and child mortality. While RSV disease burden is highest in low- and middle-income countries, most knowledge about risk factors for fatal RSV disease comes from high-income settings.

**Methods:**

Among infants aged 4 days to <6 months who died at University Teaching Hospital in Lusaka, Zambia, we tested nasopharyngeal swabs obtained postmortem for RSV using reverse transcriptase–quantitative polymerase chain reaction. Through a systematic review of death certificates and hospital records, we identified 10 broad categories of underlying medical conditions associated with infant deaths. We used backward-selection models to calculate adjusted and unadjusted risk ratios (RRs) for the association between each underlying condition and RSV status.

**Results:**

From 720 infant deaths, 6% (44) were RSV-positive, 70% were <4 weeks old, and 54% were male. At least 1 underlying condition was found in 85% of infants, while 63% had ≥2. Prematurity/low birth weight (53% [384]) and complications of labor and delivery (32% [230]) were the most common conditions. Congenital cardiac conditions were significantly associated with an increased risk of RSV infection (4%, 32; adjusted RR: 3.57; 95% CI: 1.71–7.44). No other underlying conditions were significantly associated with RSV.

**Conclusions:**

Other than congenital cardiac conditions, we found a lack of association between RSV and underlying risk factors. This differs from high-income settings, where RSV mortality is concentrated among high-risk infants. In this population, birth-related outcomes are the highest mortality risk factors. Improved neonatal care remains crucial in the fight against neonatal mortality.

Globally, acute lower respiratory infection (ALRI) is a leading cause of child mortality, and respiratory syncytial virus (RSV) is the most prevalent pathogen in children who develop ALRI [1, 2]. While the vast majority of RSV ALRI cases occur in low- and middle-income countries [3], much of our knowledge of underlying risk factors comes from high-income countries where there are well-defined groups that are at high mortality risk due to RSV. In particular, mortality risk is elevated in infants with comorbid conditions such as prematurity and underlying heart disease [4], or those experiencing risk factors such as poverty, crowding, and malnutrition [5]. Considerably less is known about the association between identifiable underlying risk factors and RSV deaths in developing countries [6].

In order to address this gap in knowledge, we conducted an analysis utilizing data from the recently completed Zambia Pertussis Infant Mortality Estimation Study (ZPRIME), a surveillance study that aimed to calculate the burden of RSV and pertussis disease among infant deaths. The present analysis focuses on infants who died after hospital admission, and for whom we were able to collect medical record data. Our aim was to identify potential risk factors associated with RSV infection among infant deaths occurring at a tertiary care referral hospital in Lusaka, Zambia. Specifically, we sought to answer the following questions: Is there an association between age and sex and RSV mortality? Do Zambian infants who die with RSV have definable medical risk factors we typically associate with RSV mortality in high-income settings? And, are RSV deaths concentrated among infants who would be defined as “high risk” for RSV mortality based on associations observed in high-income settings, or does RSV mortality occur at high rates even among infants without definable risk factors?

## METHODS

**Figure 1.**
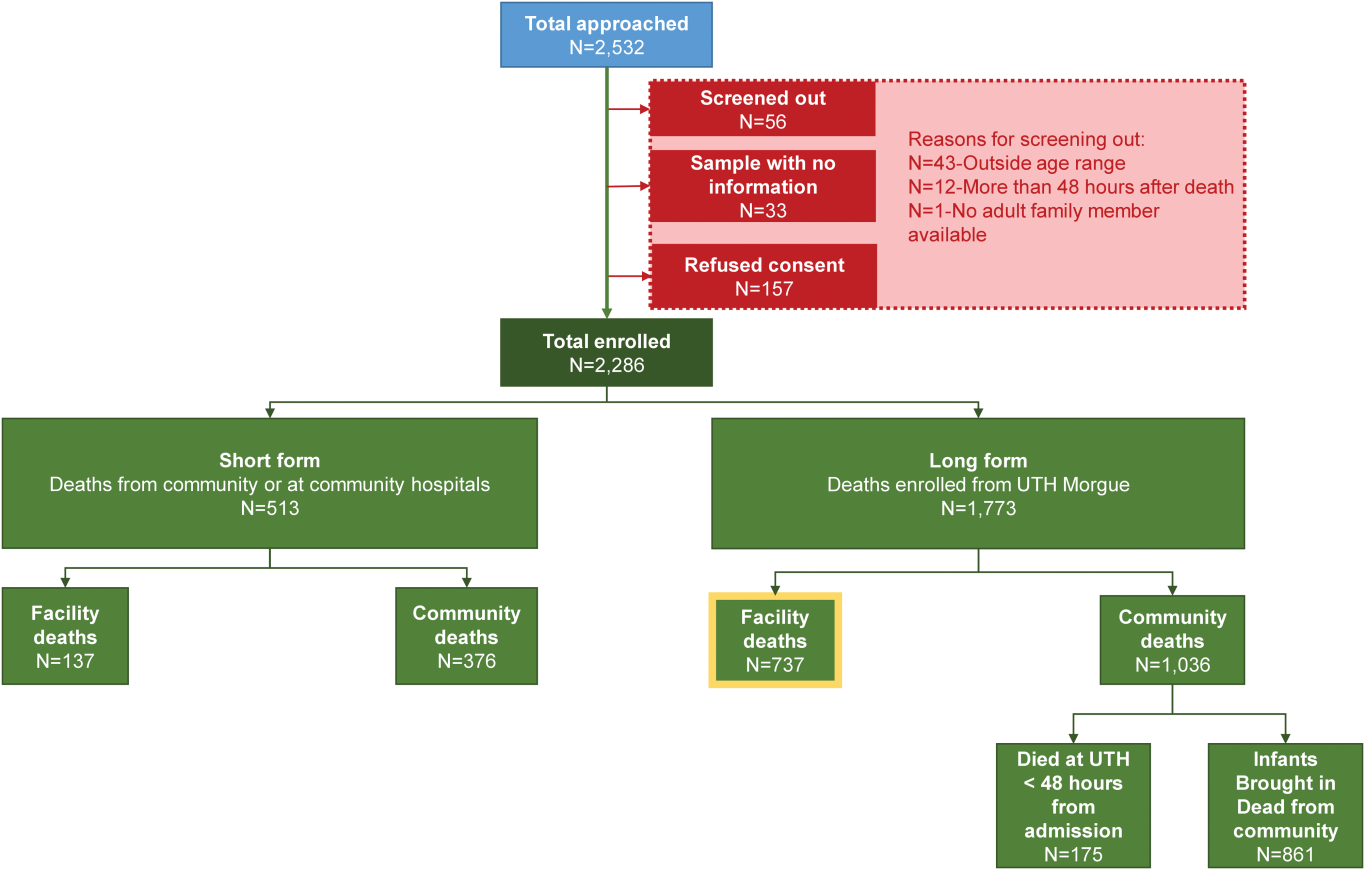
Flowchart showing the entry pathway for final enrollment into ZPRIME including the sources of noninclusion. Between the August 2017 and August 2020 ZPRIME we enrolled 2286 infants aged 4 days to <6 months. For infants enrolled from the UTH morgue, we collected “long form” data, which included demographic and clinical data that we extracted from the medical charts and/or the medical certificate of the cause of death. For this analysis, we focus on facility deaths that were enrolled between August 2017 and December 2019 for which we had collected “long form” data (highlighted in yellow). The present analysis includes ~98% of all these deaths. Abbreviations: UTH, University Teaching Hospital; ZPRIME, Zambia Pertussis Infant Mortality Estimation Study.

ZPRIME study researchers identified infants aged 4 days to younger than 6 months who died in Lusaka, Zambia, from August 2017 through August 2020. The present analysis utilizes a subset of the full cohort and includes data collected through December 2019. In Zambia, a burial cannot occur without a burial permit. Therefore, all deaths must first be cleared for burial at the medical examiner’s office at the University Teaching Hospital (UTH) or one of a select number of smaller clinics. For this analysis, we focus on deaths that occurred at the University Teaching Hospital in Lusaka, for which we had access to medical history data from the death certificates and clinical charts (Figure 1). In a separate analysis within this issue of *Clinical Infectious Diseases*, we present explanatory data pertaining to community RSV deaths (see Murphy et al in this Supplement issue).

For all deaths, researchers approached family members accompanying the body to the UTH morgue and, after obtaining informed consent, enrolled them in the study, and noted their age, sex, and date of death. We obtained postmortem nasopharyngeal (NP) samples from each infant using flocked nylon swabs (Copan Diagnostics, Murietta, CA). The swabs were placed in universal transport media on ice, transported to the microbiology laboratory on site at UTH, and stored at −80°C until RSV testing. Nucleic acid extraction was performed using the NucliSens easyMAG system (bioMérieux, Marcy I’Etoile, France), a system for automated isolation of nucleic acids from clinical samples based on silica extraction technology. Screening for RSV used a singleplex reaction specific to the dominant M protein on the virus using reverse transcriptase–quantitative polymerase chain reaction (RT-qPCR), a following the RSV protocol from the respiratory viruses branch at US CDC [7]. In order to demonstrate that the NP swab made effective contact with the respiratory mucosa, each sample run included primers/probes specific to the human constitutive enzyme RNAseP, which is expressed in all human cells (including the nasal epithelium). Its presence, therefore, validates the adequacy of the sample collection process. A positive RSV signal was defined as having a cycle threshold (CT) value of less than 40. All runs included positive and negative controls.

Using the death certificates and hospital records of deceased infants, we identified sections of these forms where conditions related to the infants’ health before death were recorded. Fields analyzed from hospital records included 2 free-text fields: “provisional diagnosis” and “clinic diagnosis or reason for referral,” and checkboxes indicating maternal human immunodeficiency virus (HIV) status, prematurity, complications of labor and delivery, low birth weight, and malnutrition. Death certificates in Zambia categorize the cause of death hierarchically with 3 causes of death. All 3 of these fields were included in our analysis. These fields are as follows: “disease or condition directly leading to death,” “antecedent cause,” and “morbid conditions giving rise to the above cause.”

We ran frequency tabulations of the data entered into these fields. The frequencies were then reviewed by the principal investigator who is a child survival expert and infectious disease specialist. He collapsed all of the underlying conditions into 1 of the 10 broad categories, as listed in Table 1. These categories were both hypothesis-generating, based on patterns seen in the data themselves, and based on historical precedent, utilizing prior knowledge of risk factors for infant mortality and respiratory disease. To confirm these categorizations, they were then reviewed by 2 additional infectious disease physicians, including a Zambian physician based in Lusaka. Often, the full-text field would list out the condition directly, but conditions were also described idiosyncratically and/or via a variety of different verbatim terms. For example, “low birth weight” was often listed using the acronym LBW, or VLBW (very low birth weight), or ELBW (extremely low birth weight), etc. Similarly, “congenital cardiac conditions” could appear as cyanotic heart disease, congenital heart disease, complex heart disease, or cardiomyopathy. It could also appear using various acronyms, or specifying certain syndromes directly (eg, Tetralogy of Fallot, often abbreviated as TOF). Infants were categorized as having prematurity in cases where the full words “prematurity” or “preterm delivery” were present. In other cases, conditions were described generically without a detailed anatomic explanation. The full list of verbatim terms and how these were collapsed into the 10 final conditions is included in Supplementary Table 1.

**Table 1.** Demographic Risk Factors, Medical Risk Factors, and Underlying Conditions by Presence of Respiratory Syncytial Virus

	RSV Status		
	Overall (n = 720)	Positive (n = 44)	Negative (n = 676)
Demographic characteristics			
Child gender			
Male	387 (53.8%)	20 (45.5%)	367 (54.3%)
Female	328 (45.6%)	24 (54.5%)	304 (45.0%)
Not recorded	5 (0.7%)	0 (0.0%)	5 (0.7%)
Age at death			
4–6 days	208 (28.9%)	6 (13.6%)	202 (29.9%)
7–13 days	186 (25.8%)	10 (22.7%)	176 (26.0%)
14–27 days	111 (15.4%)	7 (15.9%)	104 (15.4%)
1–2 months (28–60 days)	88 (12.2%)	8 (18.2%)	80 (11.8%)
3–5 months (61+ days)	127 (17.6%)	13 (29.5%)	114 (16.9%)
Median age at death (25th, 75th), days	12 (6, 38)	24 (11, 81)	11 (6, 35)
Infant aged <1 month at death	505 (70.1%)	23 (52.3%)	482 (71.3%)
Infant aged ≥1 month (≥28 days) at death	215 (29.9%)	21 (47.7%)	194 (28.7%)
Mother and/or father employed	582 (81.9%)	37 (86.0%)	545 (81.6%)
No. of people living in household, median (25th, 75th)	4 (3, 6)	4 (3, 6)	4 (3, 6)
Currently breastfeeding	644 (91.6%)	40 (95.2%)	604 (91.4%)
Place of birth			
Other	73 (10.2%)	2 (4.7%)	71 (10.5%)
Hospital/health facility	646 (89.8%)	41 (95.3%)	605 (89.5%)
Did the child ever leave the hospital prior to the fatal illness? Yes	431 (59.9%)	30 (68.2%)	401 (59.3%)
Did the child receive any immunizations? Yes	133 (18.8%)	10 (23.3%)	123 (18.6%)
Infant HIV positive: yes	53 (7.4%)	3 (6.8%)	50 (7.4%)
Nosocomial RSV			
Unambiguous	14 (31.8%)	14 (31.8%)	0 (0.0%)
Not nosocomial	10 (22.7%)	10 (22.7%)	0 (0.0%)
Potentially nosocomial	20 (45.5%)	20 (45.5%)	0 (0.0%)
Medical risk factors			
Prematurity/low birth weight: yes	384 (53.3%)	23 (52.3%)	361 (53.4%)
Complications of labor and delivery: yes	230 (31.9%)	13 (29.5%)	217 (32.1%)
Maternal HIV exposure: yes	159 (22.1%)	12 (27.3%)	147 (21.7%)
Congenital conditions: yes	111 (15.4%)	7 (15.9%)	104 (15.4%)
Hypoxic ischemic encephalopathy: yes	95 (13.2%)	4 (9.1%)	91 (13.5%)
Conditions associated with prematurity: yes	56 (7.8%)	0 (0.0%)	56 (8.3%)
Congenital cardiac conditions: yes	32 (4.4%)	7 (15.9%)	25 (3.7%)
Malnutrition: yes	32 (4.4%)	3 (6.8%)	29 (4.3%)
Down syndrome and other chromosomal defects: yes	11 (1.5%)	1 (2.3%)	10 (1.5%)
Syphilis: yes	10 (1.4%)	1 (2.3%)	9 (1.3%)
No conditions	111 (15.4%)	5 (11.4%)	106 (15.7%)

Data are presented as n (%) unless otherwise indicated. Abbreviations: HIV, human immunodeficiency virus; RSV, respiratory syncytial virus.

We then assigned underlying conditions to each infant based on the presence of keywords related to those 10 categories. Each field was analyzed independently of other fields for the same subject to avoid bias based on a full review of a particular infant’s health records. Infants could therefore be assigned 0, 1, or more underlying conditions. Demographic characteristics, such as place of birth (hospital or health facility vs other), parental employment and education, and number of people in the household, were collected via direct interview with the caretaker who accompanied the body to the morgue.

### Statistical Analyses

Descriptive statistics were calculated to determine the prevalence of each of the above conditions, demographic characteristics, and medical risk factors, stratified by RSV status. Unadjusted log-binomial regression models were used to compute risk ratios (RRs) for each underlying condition and its association with RSV. Given the study design (prospective cohort study), we feel that we were able to capture all infants at risk during the period, and therefore RRs are an appropriate measure of the risk of RSV in the population.

In order to determine which underlying factors and covariates were associated with RSV, we used a backward-selection approach. We ran 10 separate models: one for each of the underlying conditions, with the exception of conditions associated with prematurity, as there were zero RSV-positive infants with that condition, plus one for infants with none of the identified conditions. All demographic and medical risk factors shown in Table 1 were included in the original models, and the underlying condition was forced into the final model. We used a *P*-value threshold of .2 for inclusion in the final model.

Last, we ran adjusted and unadjusted models for combinations of conditions which, based on prior medical knowledge, are often related. We created 1 merged condition such that infants with either 1 or both risk factors were classified as yes and infants who did not have either of the risk factors were classified as no. The following merged conditions were used: HIV exposure and/or malnutrition, prematurity and/or congenital cardiac conditions, and prematurity and/or conditions associated with prematurity.

## RESULTS

There were 720 total facility deaths that occurred at UTH included in this analysis. Following PCR testing of the NP swabs, 6% (44) were RSV positive. Demographic risk factors, medical risk factors, and underlying conditions are summarized in Table 1. The majority of infants (70%) were younger than 4 weeks old. Fifty-four percent were male, 90% were born in a hospital or health facility, and 40% had been born at UTH and did not survive to discharge. The median household size (including the infant) was 4 people. Nineteen percent received some vaccinations.

Of the 44 RSV-positive infants, 32% (14) never left the hospital, meaning their RSV infection was nosocomial—it originated in the hospital. Ten infants left the hospital and returned less than 48 hours before passing, which leads us to believe RSV infection for these infants originated in the community. For the remaining 20 infants who left the hospital and returned for more than 48 hours before passing, the origin of their RSV infection is unknown.

Underlying conditions were identified in the majority (85%; 609) of infants, many of whom had more than 1 condition identified. More specifically, 22% (160) had 1 underlying condition, 28% (199) had 2, 23% (164) had 3, 12% had 4 or more underlying conditions. Fifteen percent (111) had no identifiable underlying conditions. The most common conditions in the sample were related to birth outcomes: prematurity/low birth weight was the most common underlying condition, present in 53% (384) of all deaths, followed by complications of labor and delivery (32%; 230). The next most common conditions were HIV exposure (22%; 159), congenital conditions other than cardiac congenital conditions (15%; 111), and hypoxic ischemic encephalopathy (HIE) (13%; 95). All other conditions—Down syndrome and other chromosomal defects, conditions associated with prematurity, malnutrition, congenital cardiac conditions, syphilis, and HIV exposure—were present in fewer than 10% of infants.

We found a few differences when comparing demographic characteristics of RSV-positive versus RSV-negative deaths. Most noticeably, infants who died due to RSV tended to be much older than infants without RSV. For example, 29.9% of RSV-negative infants had died between days 4 and 6 of life, whereas a similar proportion (29.5%) of RSV-positive deaths were aged between 3 and 5 months (RR for the association between RSV and age older than 1 month: 2.14; 95% confidence interval [CI]: 1.21, 3.78; *P =* .01). In addition, RSV-positive infants were more likely to be female (55% vs 45%; RR: 1.42; 95% CI: 0.80, 2.52; *P =* .24) and to have left the hospital and have been readmitted during the fatal illness (68% vs 59%; RR: 1.41; 95% CI: .76, 2.62; *P* = .27). Other factors, such as breastfeeding status, which was nearly universal in all infants, parental employment status, and the number of persons living in the household, were similar between the RSV-positive and -negative deaths.

Results from the adjusted and unadjusted regression models are presented in Table 2. There was a noticeable difference between RSV-positive and RSV-negative infants in the proportion with a congenital cardiac condition: it was identified as an underlying cause in 16% (7/44) of the RSV-positive infants versus 4% (25/676) in the RSV-negative infants (RR: 4.04; 95% CI: 1.95, 8.34; *P* < .001). In the final backward-selection model, congenital cardiac conditions remained significantly associated with an increased risk of RSV infection (RR: 3.57; 95% CI: 1.71, 7.44; *P* = .001). In addition, infant age at death and place of birth (hospital/health facility vs other) remained in the model at a *P*-value threshold of .2. Infants 28 days or older were significantly associated with an increased risk of RSV infection (RR: 1.95; 95% CI: 1.08, 3.50; *P* = .03), while place of birth trended toward an increased risk of RSV infection, although this association did not reach statistical significance.

**Table 2.** Associations With Respiratory Syncytial Virus (Adjusted and Unadjusted Risk Ratios)

	Unadjusted		Adjusted	
Underlying Condition	Risk Ratio (95% CI)	*P*	Risk Ratio (95% CI)	*P*
Individual risk factors				
Prematurity or low birth weight	.97 (.55, 1.72)	.920	1.30 (.71, 2.37)^a^	.393
Complications of labor and delivery	.89 (.47, 1.67)	.716	1.08 (.56, 2.07)^b^	.823
Maternal HIV exposure	1.31 (.69, 2.49)	.406	1.14 (.59, 2.17)^b^	.701
Congenital conditions (other)	1.03 (.47, 2.25)	.942	.90 (.39, 2.08)^b^	.805
Hypoxic ischemic encephalopathy	.65 (.24, 1.78)	.405	.81 (.29, 2.27)^b^	.695
Congenital heart disease	4.04 (1.95, 8.34)	<.001	3.57 (1.71, 7.44)^a^	.001
Malnutrition	1.56 (.51, 4.77)	.434	.97 (.31, 3.07)^b^	.964
Genetic syndrome	1.49 (.22, 9.86)	.680	1.30 (.20, 8.45)^b^	.783
Syphilis	1.64 (.25, 10.76)	.607	1.80 (.28, 11.83)^b^	.539
No conditions identified	.70 (.28, 1.73)	.437	.64 (.26, 1.59)^b^	.338
Combinations of related conditions				
Prematurity and/or complications associated with prematurity	.66 (.35, 1.23)	.189	.83 (.42, 1.62)^b^	.583
Prematurity and/or congenital heart disease	1.03 (.58, 1.85)	.914	1.37 (.75, 2.51)^a^	.311
HIV and/or malnutrition	1.40 (.76, 2.58)	.283	1.15 (.61, 2.16)^b^	.668

Abbreviations: CI, confidence interval; HIV, human immunodeficiency virus.

^a^Adjusted for age at death and place of birth.

^b^Adjusted for child gender, age at death, and place of birth.

Other than congenital cardiac conditions, RSV infection was not significantly associated with any other underlying conditions in adjusted or unadjusted models. In all models, infant age at death of 28 days or older remained significantly associated with an increased risk for RSV. In addition to infant age at death, location of birth remained under the *P*-value threshold of .2 for model inclusion in all models, and child gender reached the threshold for 10 out of 13 models. In all models, being born in a health facility and female gender were associated with an increased risk of RSV infection, although neither was statistically significant in any model.

While the association with RSV for many of these conditions was minimal (odds ratios were close to 1), some were associated with increased risk for RSV, while others were protective. In particular, congenital cardiac conditions, syphilis, Down syndrome and other congenital defects, and prematurity/low birth weight were all associated with a slightly increased risk for RSV; HIE and “no conditions” appeared to have a slightly protective association with RSV. HIV exposure, complications of labor and delivery, malnutrition, and noncardiac congenital conditions were neither protective nor presented an increased RSV risk.

## Discussion

The key finding in this analysis was the strong association between the presence of congenital cardiac conditions and an elevated risk of death due to RSV, an association that has been documented in multiple other studies from high-income countries. In contrast, we did not observe an association between RSV and prematurity, the latter being an oft-cited risk factor for adverse or fatal RSV infections in wealthy countries. We also observed a clustering of RSV mortality as a function of age, with a higher prevalence of RSV among older infants who died. Each of these merits further discussion.

The strong association between congenital heart disease and severe or fatal RSV has been noted for decades and should be unsurprising. The syndrome due to RSV, bronchiolitis, leads to hypoxemia due to impaired gas exchange, and it makes sense that infants who already have a pre-existing cause of hypoxemia due to cyanotic heart disease would be less resilient. It was reported by Macdonald et al [8] in 1982 and confirmed in subsequent studies [9], including a Canadian study that found Congenital Heart Disease (CHD) was associated with longer hospital stays due to RSV [10]. A recent meta-analysis looking specifically at the association of CHD with RSV concluded that infants with CHD were associated with higher risks for severe RSV-ALRI and hospitalization, and had a higher case fatality ratio [11]. Notably, that meta-analysis included studies from North America, South America, Europe, and Asia, but none from Africa. This study, therefore, contributes to the growing body of knowledge around the association between congenital cardiac conditions and RSV.

In contrast, the absence of an association between prematurity in the current study and RSV mortality is surprising and forces us to consider and perhaps reconcile this apparent exception. In high-income settings, low birth weight and prematurity are known risk factors that significantly contribute to neonatal mortality [12, 13], and existing literature links these risk factors with RSV mortality in particular [14, 15]. It is therefore notable that these conditions were not associated with RSV in the present study, even though birth-related underlying conditions were our most prevalent conditions. Specifically, approximately half of all infants who died in this cohort were listed as having prematurity and/or low birth weight. As with cardiac disease, or bronchopulmonary dysplasia, prematurity is believed to increase the risk from RSV infection due to a combination of anatomy (smaller airways being more vulnerable to mucus obstruction), chemistry (the absence or paucity of surfactant in immature lungs), and immunology (reduced transplacental transfer of maternal immunoglobulin G [IgG]). We offer several theories to explain our findings. One plausible explanation is that, in low-income settings such as this one, infants born with these birth-related comorbidities are less likely to survive long enough to contract RSV. As such, the lack of association between low birth weight and RSV, and prematurity and RSV, can be reflective of a survival bias. Our data are very consistent with this explanation. The majority of infants (70%) in our sample, which was designed as a comprehensive survey of all infant deaths at UTH, died at younger than 1 month old.

Another explanation is that it is quite difficult to define prematurity in low-income-country settings given the general absence of robust strategies to accurately measure gestational age, such as prenatal ultrasound. In that absence, the appellation of “prematurity” could be applied rather loosely by the clinicians who attended to these infants at UTH, using the term generically to describe any small or low-weight infant, even though some proportion of these low-birth-weight infants may have been full term but experienced some insult resulting in intrauterine growth retardation. That would tend to introduce an element of random misclassification, biasing associations towards the null. This theory is supported by the high proportion of infants who were described in the clinical notes as both low birth weight and premature, suggesting that the clinicians were hedging on making this distinction. Moreover, these 2 explanations are not mutually exclusive.

Immunodeficiency and HIV infection are other oft-cited risk factors for severe RSV disease. In a 2013 study of RSV hospitalizations in South Africa, Moyes et al [16] concluded that children with HIV are at greater risk of hospitalization due to RSV than children without HIV. Our data did not show a significant association between maternal HIV and RSV infection, or between child HIV and RSV infection. We did not directly access maternal health records in our study, nor did we independently confirm maternal HIV status. The lack of association between HIV infection and RSV mortality could therefore be due to incomplete reporting on maternal HIV in the child’s medical record. In addition, treatment of HIV in mothers could reduce the severity of risks passed onto the child after birth. Given that the majority of our sample died at less than 1 month old, maternal antibody could still provide some level of protection from HIV in these infants.

The strongest and most consistent association with RSV infection in our dataset was with age at death. Even after adjusting for underlying conditions, older age remained significantly associated with an increased risk of RSV infection. This polarization in age likely reflects the source of RSV acquisition. Prematurity and complications of labor and delivery were extremely common and often lead to death very early in life. It is self-evident that an infant cannot die of RSV if he/she dies first of complications related to prematurity. Rather, infants who are well enough to be discharged to their home are, paradoxically, at risk of exposure to RSV and of dying of RSV. These suppositions are supported in our data by the overall older ages of infants who died of RSV versus non-RSV deaths, and the higher proportions of RSV deaths occurring among infants who had first been discharged to the community after birth and were readmitted during their final fatal illness (RR: 1.41; 95% CI: .76, 2.62; *P =* .27).

In a low-income country such as Zambia, infants with adverse birth outcomes do not receive the same comprehensive care as they do in high-income settings, and are therefore are at higher risk for mortality [13]. We hypothesize that this also contributes to the association between RSV mortality and older age: infants who died from birth-related factors did not live long enough to contract RSV. This is borne out by our data: for the majority of conditions identified in our dataset, those who were RSV positive were older than those who were RSV negative.

### Limitations

Our study had several limitations. The present analysis is an analysis of routine clinical data collected during our surveillance study, the goal of which was to determine the overall prevalence of RSV infant mortality in Zambia. We therefore did not systematically collect the source data used for this analysis and relied on existing records used for patient care and documentation that were not built specifically to answer our questions. Both primary sources, medical records and death certificates, would most likely include information on the most relevant and proximate causes of illness and death. We therefore expect that there is underreporting of many related, less proximate conditions.

Survivor bias due to age at death was another limitation of this study. Because our study includes such young children, a plurality of them had underlying conditions related to circumstances surrounding their birth, such as low birth weight and prematurity. Due to sample-size limitations, we were unable to perform any sensitivity analyses using a sample limited to older infants. In addition, our analysis was limited to infants who died. We therefore were not able to tease out which of these underlying conditions are present in infants who survive, as well as those who do not. However, the systematic nature of our data collection, and the fact that we captured the majority of all infant deaths in the study period, provided us with a unique opportunity to comprehensively study RSV in this infant population.

### Conclusions

Our study largely showed a lack of association between underlying conditions and RSV mortality, with the notable exception of congenital cardiac disease, which was associated with a significant increase in RSV mortality. This differs from the United States and similar settings, where RSV disease is ubiquitous but RSV mortality is concentrated among infants with co-occurring risk factors [4, 5]. In this neonatal population, birth-related outcomes were the highest risk factors for mortality. Had some of these infants survived past the early neonatal period, we believe more of them would have contracted RSV. Improved neonatal care in low-income parts of the world remains the crucial factor in the fight again neonatal mortality. Once infants survive the early neonatal period, other infections, including RSV, which is among the most prevalent, remain mortality risks. Further research, with an expanded age at death past the early neonatal period, is warranted.

## Supplementary Data

Supplementary materials are available at *Clinical Infectious Diseases* online. Consisting of data provided by the authors to benefit the reader, the posted materials are not copyedited and are the sole responsibility of the authors, so questions or comments should be addressed to the corresponding author.

ciab466_suppl_Supplementary_Material

## Notes

***Supplement sponsorship.*** This supplement is sponsored by the Bill and Melinda Gates Foundation.

***Financial support.*** The ZPRIME study was made possible through the generous support of the Bill and Melinda Gates Foundation (OPP1163027).

***Potential conflicts of interest.*** All authors: No reported conflicts of interest. All authors have submitted the ICMJE Form for Disclosure of Potential Conflicts of Interest. Conflicts that the editors consider relevant to the content of the manuscript have been disclosed.
