# Supplementary material for: Association of Respiratory Syncytial Virus Infection and Underlying Risk Factors for Death Among Young Infants Who Died at University Teaching Hospital, Lusaka Zambia
Source: Clin Infect Dis. 2021 Sep 2;73(Suppl 3):S180–6. doi: 10.1093/cid/ciab466 (PMC8411249; doi:10.1093/cid/ciab466)
Supplement: ciab466_suppl_Supplementary_Material [file ciab466_suppl_supplementary_material.docx]

**Supplemental Table 1: List of conditions and associated terms**

| Underlying Condition | Associated terms |
| --- | --- |
| Conditions associated with prematurity | NEC [i.e., necrotizing enterocolitis] |
|  | Intraventricular hemorrhage |
|  | Intraventricular bleed |
|  | Spina bifida |
|  | Nectrotic bowel |
|  | Necrotising Enerocolitis |
|  | Neonatal sepsis |
|  | PPHN [i.e., persistent pulmonary hypertension of the newborn] |
|  | Persistent pulmonary hypertension |
|  | Pulmonary hemorrhage |
|  | Respiratory distress syndrome |
| Complications of Labor and Delivery | Asphyxia / Severe birth asphyxia |
|  | Birth trauma |
|  | Hypothermia |
|  | Meconium aspiration |
|  | Eclampsia |
|  | prolonged labor |
|  | Strangulated hernia |
| Congenital Cardiac Conditions | Congenital heart disease |
|  | CCF [i.e., congenital cardiac failure] |
|  | CHD |
|  | Cyanotic heart disease |
|  | Cyanosis Hypocephalus |
|  | PDA [ i.e., patent ductus arteriousus] |
|  | TEF/OA [i.e, a typographical error for Tetralogy of Fallot] |
|  | Tetralogy of Fallot |
|  | Truncus arteriousus |
|  | VSD [i.e., ventricular septal defect] |
| Congenital other conditions | Achondra Plasia |
|  | Imperforate anus |
|  | Anal stenosis |
|  | Anencephaly |
|  | Artesia repair malrotation internal volvulus reduction |
|  | Arthrogryposis multiplex |
|  | Bilateral hernia |
|  | Cleft lip and/or palate |
|  | Bilateral Tallipes Equino Varus deformities |
|  | Talipes syndrome |
|  | Birth defects |
|  | Omphalocele |
|  | CTEV |
|  | Choanal Atresia |
|  | Club foot |
|  | Colonic atresia |
|  | Gastrosciasis |
|  | Hydrocephalus |
|  | Congenital intestinal obstruction |
|  | Congenital malformations |
|  | Congenital abnormalities |
|  | Congenital hydrocephalus |
|  | Cystic Hydroma |
|  | Duodenal atresia |
|  | Encephalocele |
|  | Enlarged multicystic fetal kidney |
|  | Esophageal artesia |
|  | Freeman Sheldon syndrome |
|  | Gasroschisis |
|  | Frontal nasal encephalocele |
|  | Hirschsprings disease |
|  | Huge occipital encephalocele |
|  | Spinal bifida |
|  | ICP |
|  | Multiple digits on both upper and lower limbs, unable to open eyes |
|  | Spina bifida |
|  | Intestinal obstruction |
|  | Left leg deformity |
|  | Multiple congenital abnormalities |
|  | Myelomeningocele |
|  | Oesophageal atresia |
|  | Passing stool via the vagina since birth |
|  | Polydactyle |
|  | Post MMC Repiar |
|  | Pyloria stenosis |
|  | Schizercephalus |
|  | Shunt sepsis |
|  | Small intestine atresia |
|  | Snake bite |
|  | Tallipes Equinovarus |
|  | Esopgageal fistula |
|  | Tracheoesophageal fistula |
| HIE | HIE |
|  | HIE II |
|  | HIE with seizures |
| Low birth weight | LBW |
|  | ELBW |
|  | VLBW |
|  | Extreme low birth weight |
| Malnutrition | Acute malnutrition |
|  | Electrolye imbalance |
|  | PCP with SAM wasting |
|  | Failure to thrive |
|  | Infantile malnutrition |
|  | SAM |
| HIV exposure | PCP |
|  | Pneumosytic carini |
|  | RVD exposed |
| Prematurity | Prematurity |
|  | Apnea of prematurity |
|  | Low birth weight and prematurity |
|  | Gross prematurity |
|  | Pre-term delivery |
| Syndromic baby | Dysmorphic baby |
|  | Chromosomal abnormalities |
|  | Congenital syndromic defects |
|  | Downs syndrome |
|  | Freeman Sheldon syndrome |
|  | Trisomy 21 |
|  | Tuscany, VSD |
| Syphilis | Congenital shyphilis |
|  | Congenital Louis |
|  | RPR positive |
